# Assessment of the usefulness of integrated disease surveillance and response on suspected ebola cases in the Brong Ahafo Region, Ghana

**DOI:** 10.1186/s40249-015-0051-3

**Published:** 2015-04-10

**Authors:** Kofi Issah, Kennedy Nartey, Richard Amoah, Emmanuel George Bachan, Jacob Aleeba, Enuamah Yeetey, Timothy Letsa

**Affiliations:** Ghana Health Service, Regional Health Directorate, P.O. Box 145, Sunyani, Brong Ahafo Region Ghana; Kintampo Health Centre, Ghana Health Service, Kintampo, Brong Ahafo Region Ghana

**Keywords:** Ebola, Disease surveillance, Brong Ahafo, Ghana

## Abstract

**Background:**

This study assessed the quality, core and support functions of the integrated disease surveillance and response (IDSR) system relating to 18 suspected cases of Ebola virus disease (EVD) in the Brong Ahafo Region, Ghana.

**Methods:**

Data was collected on selected indicators of the surveillance system relating to 18 suspected cases of EVD, from epidemiological week 19 to 45 of 2014. We conducted in-depth interviews with seven medical directors and two district directors of health services, and also reviewed documentation on the implementation of the core, support and quality functions of the IDSR system. We also monitored news in the media and rumours about EVD within the community as well as in health facility surveillance registers.

**Results:**

The study identified gaps in the implementation of IDSR relating to 18 suspected cases of EVD. Health staff heavily relied on haemorrhage as the only symptom for detection of suspected EVD cases. Twelve blood samples and a swab of secretions from the mouth of the thirteenth patient (who died) tested negative for EVD using PCR assay in laboratory confirmation. The blood samples of three patients were discarded, as they did not fit the case definition for suspected cases, whilst two refused for their blood samples to be taken.

The community-based surveillance (CBS) system has not been given a prominent role in EVD surveillance and response, as demonstrated by CBS volunteers and health staff not receiving any training in these processes.

There was intense public interest in EVD in August and September 2014. That interest has since waned for reasons that have to be formally ascertained. Unfounded fear of and anxiety about EVD still remain challenges due to a lack of in-depth knowledge about the disease in Ghana.

**Conclusion:**

Ghana has been one of the pioneers in the implementation of IDSR in Africa. Despite this, gaps have been identified in the implementation of IDSR relating to EVD in the Brong Ahafo Region. To address these gaps, the CBS system has to actively partner with health facility surveillance to achieve effective IDSR in the region.

**Electronic supplementary material:**

The online version of this article (doi:10.1186/s40249-015-0051-3) contains supplementary material, which is available to authorized users.

## Multilingual abstracts

Please see Additional file 1 for translations of the abstract into the six official working languages of the United Nations.

## Background

The outbreak of the Ebola virus disease (EVD) in West Africa has presented problems for disease surveillance systems in the sub-region, including in Ghana. The nature of the outbreak is unprecedented [1] and has placed Ghana among the 15 countries at risk of an outbreak [2]. The health systems of these countries, with their numerous problems and limitations, have no prior experience in dealing with any outbreak of viral haemorrhagic fever (VHF) on this scale. The fact that only 12 countries have the requisite capacity to conduct laboratory confirmation of EVD within the context of IDSR for priority disease intervention show the limitations of surveillance systems in Africa, including those in West Africa [3].

Most preparedness and response measures in countries such as Ghana which are unaffected by EVD have been facilitated by ‘international strengthening teams’ deployed by the World Health Organization (WHO) to support these countries assess and improve their operational readiness for EVD to the greatest degree possible [4].

Since 1998, the WHO Africa Region (WHO AFRO) adopted a strategy known as integrated disease surveillance and response (IDSR) aimed at strengthening public health surveillance and response to priority infectious diseases at district level [5]. The IDSR process integrates surveillance with laboratory support, and translates surveillance and laboratory data into specific public health action. Just like other disease surveillance strategies, IDSR has five components that can be monitored or evaluated using key indicators to assess their effectiveness (namely their structure, core functions, priority diseases for surveillance, surveillance quality and support functions) [6].

However, the effective implementation of this strategy in Africa is dependent on the combined implementation of all strategic components of IDSR to include strong coordination, efficient communication, laboratory capacity for case confirmation and training of disease control officer, public health nurses, community health nurses, field technicians etc. The availability of sustained and integrated funding for training activities is also critical [7].

Ghana started implementing IDSR in 2002 [8] and presently has 20 priority diseases, which include VHFs (EVD and others) under surveillance, with reports being sent to the national surveillance department on a weekly basis [9]. The IDSR strategy in the Brong Ahafo Region of Ghana consists of a community-based surveillance (CBS) system, which includes 2,928 volunteers reporting unusual health events from 3,292 communities, and a facility-based surveillance system made up of 665 health facilities. Since 2002, this surveillance strategy has been at the forefront of guinea worm and poliomyelitis eradication efforts, and is used for responding to diseases of epidemic potential including VHFs, cholera and meningitis. The data from this surveillance system is amongst others entered into a district health information management system (DHIMS) for storage and to be used by health managers to aid them in decision-making relating to disease control and surveillance.

Since March 2014, when the WHO declared the EVD outbreak [9], the Government of Ghana and its health sector has announced and rolled out plans to prevent and respond to any outbreak in the country. However, there have been concerns raised by various stakeholders, such as the Ghana Medical Association, that the country is not adequately prepared to handle an EVD outbreak should one occur [10].

According to the WHO, a country like Ghana, which has no reported cases of EVD, has to put in place an alert level surveillance system at major land border crossings with already affected countries, airports, capital cities and major health facilities [11]. It is therefore expected that IDSR in Ghana and Brong Ahafo should enable public health officers to identify an outbreak sooner and be confident about which communities and areas need interventions in case of an EVD outbreak [12].

Though at risk for EVD, Ghana [2] has not yet reported a confirmed case, but since March 2014 has started preparatory activities in all regions including Brong Ahafo towards forestalling or managing a potential outbreak. These activities have included raising public awareness about the disease, health worker capacity-building in contact tracing and case management, and distribution of resources including personal protective equipment (PPE) to designated health facilities.

The goal of surveillance is early detection of cases and outbreaks, rapid investigation and early laboratory confirmation of disease as case fatalities, especially for EVD, have ranged between 25% and 90% during outbreaks [13]. It is therefore critical for outbreak detection and response that epidemiological data and methods are properly utilised, not only by surveillance and response teams at district, regional and national levels, but also by other stakeholders. Unfortunately there is little evidence that the data are used by higher level technical and political leaders to prioritise and plan more effective prevention programmes and to allocate resources for improved prevention [8].

Because Ghana and Brong Ahafo have no prior experience with EVD, the CBS component of IDSR has to be given a prominent role as it helps to provide an active surveillance system. In the case of EVD, it is the communities that are best placed to detect and monitor any suspected case, mobilise themselves for action and make requests for assistance. Furthermore CBS is intended to improve public health surveillance and link communities with health facilities [14].

This study assessed the level of implementation of the quality, core and support functions and identified the gaps of the IDSR system relating to 18 suspected cases of EVD in the Brong Ahafo Region, Ghana.

## Methods

### Study area

**Figure 1:**
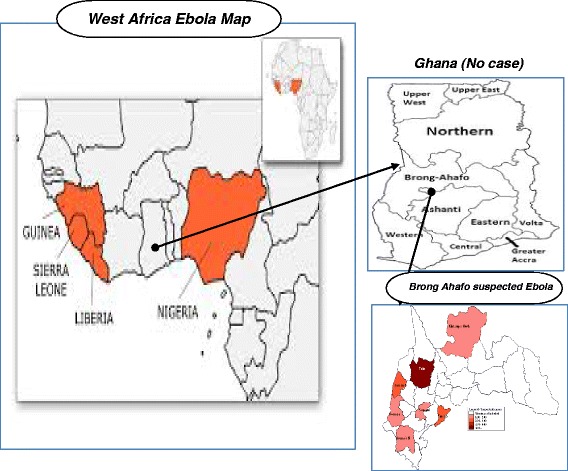
**Map showing the location of the Brong Ahafo Region in relation to Ghana, and West African Countries where cases of EVD have been recorded.**

The Brong Ahafo Region lies geographically in the middle of the Republic of Ghana. It has a population of approximately 2.5 million (see Figure 1). The vegetation in the region forms part of the forest belt of Ghana, and hunting and sale of bush meat are major activities. There is a bat colony (some of these bats have been found to harbour antibodies of the Ebola virus) at Buoyem caves (a popular tourist attraction) located in the central part of the region [15].

Ill health and death of members in any community in the region are surrounded by cultural rites and rituals that involve close contact with the body and possibly fluids of the sick or dead persons. The causes of many diseases including infectious ones are shrouded in superstitious beliefs and community members are required by tradition to visit or help nurse the sick.

Except for outbreaks of cholera in 2012 [16] and in 2014 [9], as well as isolated cases of cerebrospinal meningitis, the region has not, in recent times, faced a major outbreak of any bacterial or viral disease (including VHFs) with epidemic potential.

There are 665 health facilities in the region, including 29 hospitals with 1,200 beds. The 29 hospitals have wards, known as fever or isolation wards, where between four and 10 beds are reserved for patients with infectious diseases such as tuberculosis (TB) and yellow fever.

The isolation wards are also where patients are admitted and cared for during outbreaks of diseases with epidemic potential such as meningitis and cholera. Except for in a few instances, staff manning these wards does not have adequate training in infection prevention practices.

### Study design and data collection

This study was longitudinal in design and involved the collection of surveillance data under the IDSR strategy relating to 18 suspected cases of EVD that occurred between epidemiological weeks 19 and 45 (the Ghanaian National Disease Surveillance Unit produces a weekly bulletin to report on priority diseases under surveillance throughout the year [17]) of 2014. This period was chosen because it coincided with when the first suspected EVD case was detected and when a stakeholder called a meeting for the formal training of contact tracing and clinical case management teams (these have been absent since activities on EVD started in July 2014) for the region and districts. We assessed IDSR using selected indicators of the core and support functions and quality of surveillance systems relating to EVD through in-depth interviews with health managers and a review of documentation.

In-depth interviews were conducted with the medical directors of seven hospitals that had been required, based on geographical location or level of functionality, to designate places for isolation wards for suspected EVD cases. In addition, we interviewed two district directors of health services who had over 20 years experience in the health system and were conversant with the start of IDSR in the region approximately 10 years ago.

The elements of the core functions assessed were case detection, registration, confirmation and reporting of cases, as well as preparedness and response to the suspected cases. The elements of the support functions assessed were standards and guidelines, training, communication, coordination, resources, monitoring and evaluation. The quality of the system was assessed using the indicator for timeliness of reporting suspected cases to the regional surveillance unit (RSI).

A checklist was used to monitor rumours and news about EVD in the 27 district health systems, on the internet (one website namely www.ghanaweb.com), in newspaper articles of two government-owned national dailies (*Daily Graphic* and *Ghanaian Times*), and from radio announcements, news and discussions by members of the public on four local FM stations.

### Data entry and analysis

The data collected on the 18 suspected cases was put on a line list and entered into an Excel worksheet (Microsoft 2010), with analysis then carried out. The responses from the health managers were recorded in accordance with the component of the surveillance system for which answers were sought.

A tally sheet was used to record the number of news items in the various media and on which aspect of EVD it focused, acknowledging the reaction of members of the public. The news items selected covered the period from August to November 2014, and included any rumours, public educational messages, announcements, news bulletins or discussions about EVD and Brong Ahafo. Items on EVD that did not specify Brong Ahafo were excluded. We also reviewed surveillance system documents for rumours. This data was manually analysed based on the frequency of news items or rumours and the subject area of focus.

Permission and clearance for the study was sought and subsequently granted by the Regional Health Research Unit. Except where necessary, the data in the study has been de-identified and client anonymity has been ensured.

## Results

In the period (epidemiological week 19–45) during which we gathered data, 18 suspected cases of EVD were reported; 17 were reported to the outpatient departments (OPDs) of six district hospitals and the regional hospital, and one was found dead near a public lorry park. All 18 cases had bleeding from one part of the body as the common symptom suggestive of EVD. Fourteen of the suspected cases were male, with the ages of all cases ranging from four to 54 years.

We found that none of the 17 cases that reported at hospitals were promptly identified as suspected EVD cases on the OPD history table. Instead, this was done after they had undergone various medical and nursing procedures that demanded possible bodily contact with health staff and other patients (in one instance, a health worker decided to take a blood sample for testing after he/she conducted a lengthy procedure of placing nasal packs to stop epistaxis in a patient).

The medical directors of the seven hospitals confirmed that there was no indication that guidelines and case definitions were being used to detect suspected EVD cases at the OPDs as the first point of call in their hospitals.

The two district directors of health services claimed they knew about IDSR and how it was started in the region, and that it was an essential strategy to respond effectively to priority diseases under surveillance in Ghana. They were, however, worried that the situation pertaining to EVD was different. One of the district directors remarked:

*‘It looks like we are creating a different surveillance system for EVD because five months after the announcement of the outbreak, things are still being done from the top and we do not even have the knowledge of how to deal with a suspected case should we come across one.’*

A total of 15 blood samples from the suspected cases and one mouth swab from the deceased case were collected for laboratory investigations at the Noguchi Memorial Institute for Medical Research (NMIMR) (designated laboratory for laboratory confirmation of EVD and other VHFs). Thirteen samples (72%) were finally transported to the laboratory. Twelve blood samples and the mouth swab from the deceased patient tested negative for EVD (see Table 1) using PCR assay in laboratory confirmation at the NMIMR. The remaining three blood samples were discarded upon reassessment by medical officers within 24 hours as the symptoms exhibited by the patients did not fit the case definition for suspected EVD cases. The remaining two patients did not have their blood samples taken; one refused citing cultural reasons, whilst the other absconded after being told he was going to have to give a blood sample for EVD.

**Table 1:** **Performance of IDSR in relation to 18 suspected cases of Ebola Virus Disease in the Brong Ahafo Region**

**Component of surveillance system**	**Element**	**Indicator**	**Response**	
			**Yes**	**No**
**Core functions**	1. Case Detection	No. of hospitals using case definitions to detect cases at outpatient departments (Total n = 29)	0	29
	2. Case Registration	No. of hospitals using case based forms to register cases	8	21
		No. of Suspected cases recorded in DHIMS 2 database	3	15
		No. of hospitals recording cases in DHIMS 2	2	27
	3. Case Confirmation	No. of hospitals which have collected blood samples from suspected cases of EVD	7	22
		No. of suspected patients with blood samples or swab collected for EVD	16	2
		No. of samples confirmed negative	13	0
	4. Case Reporting	No. of hospitals that have reported cases to the District Health management Team Surveillance units	7	22
	5. Epidemic Preparedness	No. of districts with plans (Hospital plan inclusive)Total n = 27	27	0
		No. of hospitals with designated isolation facilities	7	22
		No. of hospitals with available IEC materials	29	0
	6. Response and control	No. of Districts with functional Epidemic preparedness committees	27	0
		No. of hospitals with Personal protective Equipment(PPE)	20	9
		No. of hospitals with non contact thermometers for patients	7	22
Support functions	1. Standards and guidelines	No. of hospitals with standards and guidelines for surveillance of EVD	20	9
		No. of hospital laboratories with SOP for collection, packaging and transport of specimen to national laboratory	1	28
	2. Training	No. of districts with staff trained in IDSR of surveillance for EVD	0	27
		No. of districts with community based surveillance volunteers trained in IDSR or surveillance for EVD	0	27
	3. Resources	No. of districts with budget line for EVD in surveillance plans	27	0
Quality	1. Timeliness	1. No. of suspected cases of EVD reported to regional surveillance unit within 24 hours	7	11

Only three of the 18 suspected cases were recorded in the DHIMS database (one case in week 20 and two cases in week 26), while only seven were reported to the RSI within 24 hours of being seen at the OPDs. In three instances, the RSI obtained information about the suspected cases due to rumours on the radio from two local FM stations, rather than through the district disease surveillance units.

Only seven out of the 29 hospitals in the region reported and investigated EVD cases despite the fact that IDSR guidelines and case definitions of EVD were sent to them by the RSI.

In terms of the quality functions of the surveillance system, seven suspected cases (38.8%) were reported to the RSI within 24 hours of detection.

The seven medical directors and two directors of health services informed the research team that all 27 districts in the region were instructed to draw up EVD preparedness and response plans, which included reviving their epidemic management committees. They were, however, waiting for funding for almost six months after the EVD outbreak was declared to put in place activities towards strengthening the IDSR system and to carry out its core and support functions.

The seven hospitals had designated places as isolation wards for suspected EVD patients, however, two hospitals had initial problems as staff wanted the isolation wards to be as far away as possible from the other wards for fear of contracting EVD. This resulted in the EVD isolation wards in those two hospitals being situated close to the mortuaries.

Five hundred pieces of PPE and 20 non-contact thermometers were received from the Ministry of Health and distributed to health facilities including those at international border crossings with Cote d’Ivoire. Newmont Ghana (an international gold mining company in Brong Ahafo) also donated an assorted quantity of PPE and other resources for use by the clinical case management teams. Twenty hospitals reported having at least five sets of PPE and seven had at least one non-contact thermometer to screen suspected cases at the OPDs.

Information, education and communication (IEC) materials were sent to all 29 hospitals and 27 health management teams.

All directors interviewed said the Public Health Unit of the Regional Health Services had officially informed them to intensify surveillance for EVD. None of them or their staff had been given any formal training or refresher training in IDSR, but they had attended sensitisation meetings on EVD held for stakeholders.

Formal training was yet to be conducted for a 15-member regional clinical case management team and a 16-member surveillance and contact tracing team. None of the 2,928 CBS volunteers in over 3,000 communities received any formal training or sensitisation on how to detect and report suspected EVD cases and help in contact tracing if cases tested positive.

Communication facilities have been adequate to support the surveillance system. Within 24–48 hours of receipt of the blood samples, the NMIMR provided feedback on the results via email or on the phone.

In terms of support functions, a coordinating committee made up of various stakeholders under the chairmanship of the regional minister was set up to oversee the whole surveillance and response procedure to any possible outbreak. However, for the period under assessment, no formal monitoring and supervision process was conducted by the health system and stakeholders to assess the effectiveness of the IDSR relating to EVD surveillance in the region.

There were no rumours of a suspected case of EVD officially logged in the rumour register of any health facility in the region as health staff verbally reported to the next level but failed to document it. Furthermore, a review of the documentation of the CBS registers also showed that no rumours were reported or logged in.

The media and the general public used the hospitals as their main source of information about suspected cases. There were eight articles on EVD and Brong Ahafo written in the period from August to September 2014, sourced from the main website (www.ghanaweb.com). The Ghana News Agency subsequently culled these.

One article included a story on a scare in the region due to the bats in the Buoyem caves carrying antigens of EVD, whilst another announced the death of a suspected case at a public lorry park. Two articles focused on the state of preparedness of the region to adequately respond to any potential outbreak of EVD and another two were on the results of laboratory tests for EVD carried out on the blood samples sent to the NMIMR. One article was on advocacy, urging all stakeholders in the region to adopt effective strategies and collaborate closely to prevent and effectively respond to any EVD outbreak. The last article was on assurances by the Director of Public Health of the Ghana Health Service to the people of the region that the bats at Buoyem carried antibodies and not antigens for EVD and urging the population not to panic. After September 2014, no article was sighted on the website in connection with EVD and Brong Ahafo. The two dailies published only one of the stories that was sourced on the internet.

Between July and September 2014, public education through the radio via jingles and announcements occurred on a daily basis. Each FM station, on at least a weekly basis, had a slot for discussions and phone-ins to deliberate on public reactions and preparedness to EVD in the region.

Subject areas that attracted lots of comments were on the state of preparedness of the region, fear and stigmatisation against suspected cases of EVD and what communities were to do in the event a suspected case was found in their community.

It was also found that the FM radio stations in their quest to intensify public awareness about the disease sought interviews with health facility managers. Judging from the reactions during the phone-ins, these interviews did little to reduce the fear, stigma and anxiety the public had about EVD. The medical director of one hospital said that clients refused to use services at its OPD for a whole day for fear of contracting EVD due to the presence of a suspected case at the same OPD the previous day.

The donning of PPE by health staff in order to take blood samples from patients was the single most notable thing that caused fear and apprehension amongst patients tagged as suspected cases, their relatives and members of the public.

## Discussion

An effective disease surveillance system is a prerequisite to prevent or respond to an EVD outbreak in a country such as Ghana, which is yet to report a confirmed case of the disease. The unprecedented nature of this outbreak [1] demands that the implementation of the core and support functions of the surveillance system produce information for decision-making to reduce the impact of any outbreak on communities and their livelihoods.

Ghana was one of the first countries in Africa, together with Tanzania, to implement IDSR in the region [18], which is supported by success stories of no cases of wild poliovirus recorded since 2008 [19] and a process towards certification as being guinea worm free since 2014 [20].

In this study, we acknowledge the limitations of the methodology and the potential of bias. However, we proceeded with the study in order to document the activities surrounding the surveillance and response to 18 suspected EVD cases. Despite the limitations in our methodology, one factor that guided us was our desire to find home-grown answers and to revamp the country’s IDSR system to deal with a possible EVD outbreak.

The authors have no cause to doubt that the surveillance system in the region would have reacted any differently to a disease for which it has no experience dealing with. Another area in which we acknowledge weakness is the fact that the responses of interviewees could have been greatly influenced by the public debate and reactions in Ghanaian news media to a possible EVD outbreak.

This study identified gaps in the core, support and quality functions of the IDSR system in the region. It found that the over dependence of health staff on bleeding (haemorrhage) as the only symptom from which to classify a suspected EVD case and non-utilisation of case definitions are of serious concern in outbreaks as most infected patients do not show haemorrhagic symptoms. Furthermore, the inadequate infection control precautions/barriers to nursing procedures in most of the health facilities means that any outbreak not detected and reported early can dramatically amplify [13], with a single positive case possibly infecting scores of people before being laboratory confirmed.

The focus on haemorrhage as the primary and only symptom to diagnose a suspected EVD case could possibly explain why out of the 29 hospitals, a number of health centres and in the 3,000+ communities, only seven hospitals reported suspected cases in the study period.

The registration of suspected EVD cases suffers the same fate as that of other priority disease under surveillance, with only three out of 18 cases recorded in the DHIMS database. Poor documentation of suspected cases for a disease such as EVD, in which the region has no experience, not only robs the system of vital data for effective decision-making but also hampers efforts to maintain public confidence in the disease surveillance system.

The timely collection, transport and testing of blood samples (72% of cases) and prompt receipt of test results shows the essential role laboratory support plays in the implementation of IDSR in the region. This can possibly have a positive effect of boosting the confidence of both health staff and the general public in the surveillance system and efforts of health authorities to respond to EVD in Ghana.

The level of preparedness by the designation of seven isolation facilities, and the distribution of IEC materials, PPE and non-contact thermometers has not been matched by the training of the health staff and CBS volunteers. This piecemeal approach to IDSR that does not give prominence to early formal training of health personnel and volunteers gives room for eventual sub-optimal performance of the surveillance system.

It has been recommended by other authors that training should be combined with the implementation of other strategic components of IDSR. Of critical essence is the availability of sustained and integrated funding for training activities [7].

Training should have been conducted very quickly at the beginning in order to prepare volunteers to adequately carry out community sensitisation and accurately record any community alert or suspected EVD case. Yet more than eight months after the WHO declared the epidemic, no formal training has been provided to the CBS volunteers. This lack of training has probably contributed to the non-recording of any rumours associated with EVD by any of the volunteers.

With Ghana being one of the pioneering countries in IDSR in Africa [16], it is unfortunate that its Brong Ahafo Region has not utilised its experience to respond effectively to the 18 suspected EVD cases and in the process the CBS system has totally been forgotten. This needs to be reversed as it is the CBS system which helps to provide an active surveillance system because communities are best placed to detect and monitor any suspected case of EVD, mobilise themselves for action and make requests for assistance during an outbreak [14]. Secondly, it is trained and dedicated volunteers who will be actively involved in contact tracing which is a critical element in averting spread of EVD to other communities.

The intense media attention given to EVD in August and September could have improved the public awareness about EVD though this has not been formally documented. The diverse nature of news items and discussions were, however, not sustained probably due to lack of funding or the proverbial Ghanaian shifting of interest onto more sensational headlines in the political sphere.

In a society that is apprehensive and ready to stigmatise patients with diseases of which they have little understanding, EVD joins HIV and TB on the list of the three most feared diseases. Furthermore, the donning of PPE and picking blood samples from patients with little concern for privacy adds to the already existing fear, mistrust and stigmatisation of patients amongst a public that has a phobia for diseases in which bleeding is a major symptom. One of the patients from whom a blood sample was taken remarked:

*‘I have seen pictures on television of people wearing these dresses (PPE) and treating or burying people sick from Ebola. What type of disease is this that everything including the way the doctors dress reminds one only of death?’* [male suspected case, 26 years old].

It is therefore not surprising that another patient, upon seeing the health staff dressed in PPE, refused to give a blood sample, whilst another simply absconded from the hospital. This could be averted by ensuring more privacy by having samples taken at places away from the prying eyes of the public. Effective counselling provided to patients and their relatives and ensuring confidentiality of the process are two other ways in which this issue can be addressed.

## Conclusion

Ghana finds itself in a critical situation of having to conduct surveillance and response to a disease in which its health system has no experience. Despite the vast experience of Ghana and Brong Ahafo in implementing IDSR, significant gaps relating to surveillance and response to EVD remain.

Apart from improving the implementation of the core, support and other functions of IDSR, the CBS system has to be urgently brought into the mainstream of surveillance activities for EVD and other diseases of epidemic potential.

The documentation of progress and gaps gives an opportunity to implement interventions to build a robust and sustainable IDSR system. This is of special significance as Ghana and the Brong Ahafo Region are yet to record their first confirmed case of EVD.

## Additional file

Additional file 1:
**Multilingual abstracts in the six official working languages of the United Nations.**

## Abbreviations

CBS: Community-based surveillance
DHIMS: District health information management system
EVD: Ebola virus disease
IDSR: Integrated disease surveillance and response
IEC: Information, education and communication
NMIMR: Noguchi Memorial Institute for Medical Research
OPD: Outpatient department
PPE: Personal protective equipment
RSI: Regional surveillance unit
TB: Tuberculosis
VHF: Viral haemorrhagic fever
WHO: World Health Organization
WHO AFRO: World Health Organization Africa Region
