# Supplementary material for: Assessment of the usefulness of integrated disease surveillance and response on suspected ebola cases in the Brong Ahafo Region, Ghana
Source: Infect Dis Poverty. 2015 Apr 10;4:17. doi: 10.1186/s40249-015-0051-3 (PMC4397731; doi:10.1186/s40249-015-0051-3)
Supplement: Additional file 1: — Multilingual abstracts in the six official working languages of the United Nations. [file 40249_2015_51_MOESM1_ESM.pdf]

Translation of the abstract into the six official working languages of the United Nations

تقييم مدي فائدة مراقبة الأمراض و الاستجابة لحالات الاشتباه في مرض الإيبولا في منطقة برونج أهافو غانا.

اسحق كوفي ونارتي كينيدي و أماها ريتشارد وباتشان جورج عمانويل و أليبيا جاكوب ويتي إينيوماها و ليستا تيموثي.

تلخيص

نبذة تاريخية: تقوم هذه الدراسة بتقييم جودة وجوهر وظائف الدعم لنظام المراقبة الشاملة للأمراض و الاستجابة(IDSR) المتعلق بالاشتباه في إصابة 18 حالة بفيروس الإيبولا (EVD) في منطقة برونج أهافو غانا.

المنهاجية: تم جمع البيانات من المؤشرات المختارة لنظام المراقبة المتعلق بالحالات 18 المشتبه بإصابتها ب EVDمن أسابيع تفشي الوباء الأسبوع رقم 19 إلى 45 من عام 2014. وقد قمنا بإجراء مقابلات متعمقة مع سبع مدراء طبيين وإثنان من مدراء المناطق للخدمات الطبية كما قمنا أيضا بمراجعة مستندات التوثيق الخاصة بتطبيق وظائف الدعم الأساسية و الجودة لنظامIDSR. كما قمنا بمتابعة الأخبار في الأنباء والشائعات حولEVD داخل المجتمع وكذلك في المنشآت الصحية المسجلة للمراقبة.

النتائج: وقد حددت الدراسة فجوتان في تطبيق نظام IDSR المتعلقة بحالات الاشتباه 18 في الإصابة بمرض EVD. وقد أعتمد الطاقم الصحي بدرجة كبيرة على النزيف على أنه العرض الوحيد للاشتباه وتحديد الإصابة بمرض EVD. وقد كانت نتائج تحليل أثني عشر عينة دم ومسحة من إفرازات الفم من الثلاث عشر مريض (المتوفين) سلبية لفيروسEVD باستخدام باقة PCR في المعمل للتأكيد. وقد قررنا عدم الاعتداد بعينات ثلاث من المرضى حيث إنها لم تطابق تعريف الحالات المشتبه بها بينما تم رفض أثنان إعطاء عينات من دماؤهم. ولم يتم إعطا نظام المراقبة المجتمعي CBS دور بارز في مراقبة EVD والاستجابة كما أوضح المتطوعين في نظام CBS ولم يتلقى الطاقم الطبي أي تدريب على هذه العمليات. وقد كان هناك اهتمام جماهيري بالغ بفيروس EVD في أغسطس وسبتمبر 2014. وقد خبا هذا الاهتمام لأسباب يجب تأكيدها رسميا. ولايزال الخوف والقلق غير المبررين من فيروس EVD بسبب نقص المعرفة عن المرض في غانا.

الخاتمة: كانت غانا أحدى الدول الرائدة في تطبيق نظامIDSR في إفريقيا. وعلى الرغم من وجود فجوات تم تحديدها في تطبيق نظامIDSR المتعلق بفيروس EVD في منطقة برونج أهافو غانا. ويجب أن يتكامل نظام CBS بصورة فعالة مع منشات المراقبة الطبية لتطبيق نظام IDSR بصورة فعالة في المنطقة.

Translated from English version into Arabic by Mohamed Gaafar, through

TRANSLATORS WITHOUT BORDERS

加纳·布朗阿哈福地区埃博拉疑似病例综合疾病监测与响应的实用性评估

Issah Kofi, Nartey Kennedy, Amoah Richard, Bachan George Emmanuel, Aleeba Jacob, Yeetey Enuamah and Letsa Timothy

摘要

引言：本研究基于加纳·布朗阿哈福地区 18 例埃博拉病毒病（EVD）疑似病例，评估相关综合疾病监测和响应（IDSR）系统的质量、核心和支持功能。

方法：本研究搜集了与 18 例 EVD 疑似病例相关的监测系统部分指标数据，时间是 2014 年该病流行的第 19 至 45 周。我们对七位医疗主管和两位区域医疗服务主管进行了深入访谈，也查阅了 IDSR 系统的核心、支持和质量功能实施的相关文件，还跟踪了新闻媒体、社区及卫生设施监测登记处关于 EVD 的新闻和传闻。

结果：研究发现，IDSR 在 18 例 EVD 疑似病例的执行过程中存在一些不足。卫生工作人员在诊断 EVD 疑似病例时严重依赖出血症状，并把它做为确诊 EVD 的唯一症状。在实验室确认中，有 12 份血样和第 13 例患者（已逝）口腔分泌物拭子经 PCR 检测为 EVD 阴性。因不符合疑似病例的定义，有 3 位患者的血样未检测，另有两位患者拒绝血样采集。在 EVD 监测与响应中，以社区为基础的监测 (CBS) 系统未受到重视，因为 CBS 志愿者和卫生工作人员在监测和响应过程中未接受任何相关训练。2014 年 7、8 月份正是高度关注 EVD 的时候，但这种关注随即消退，原因还有待明察。除此之外，加纳民众由于缺乏对 EVD 的深入了解，对该病产生毫无根据的恐惧和焦虑仍将是一大挑战。
结论：加纳是非洲实施 IDSR 的先驱之一。即便如此，布朗阿哈福地区在实施关于 EVD 的 IDSR 过程中仍存在一些不足。为了弥补这些不足，有效发挥 IDSR 在该地区的作用， CBS 系统必须主动与卫生设施监测进行合作。

Translated from English version into Chinese by Chen Jin, edited by Yin Jian-hai, through

CHINESE CENTER FOR DISEASE CONTROL AND PREVENTION
NATIONAL INSTITUTE OF PARASITIC DISEASES

Évaluation de l’utilité de la surveillance et de la réponse intégrées à la maladie dans des suspicions de cas d’Ébola dans la région de Brong Ahafo, au Ghana

Issah Kofi, Nartey Kennedy, Amoah Richard, Bachan George Emmanuel, Aleeba Jacob, Yeetey Enuamah et Letsa Timothy

Résumé
Contexte : Cette étude évalue les fonctions de qualité, centrales et de soutien du système de surveillance et de réponse intégrées (SRRI) en relation avec 18 cas suspects de fièvre d’Ébola dans la région de Brong Ahafo, au Ghana.
Méthodes : Nous avons recueilli des données sur certains indicateurs du système de surveillance concernant 18 cas suspects de fièvre d’Ébola au cours des semaines épidémiologiques 19 à 45 de 2014. Nous avons mené des entretiens approfondis avec sept directeurs médicaux et deux directeurs des services sanitaires de district et examiné la documentation relative au déploiement des fonctions centrales, de soutien et de qualité du système de SRRI. Nous avons également surveillé les actualités dans les média et les rumeurs de cas d’Ébola dans la communauté et dans les registres de surveillance des services de santé.
Résultats : L’étude a identifié des lacunes dans l’implémentation du système de SRRI en relation avec 18 cas suspects de fièvre d’Ébola. Le personnel de santé s’est fortement basé sur le seul symptôme des hémorragies pour détecter les cas suspects. Vingt échantillons de sang et un écouvillonnage des sécrétions buccales d’un treizième patient (décédé) ont donné un résultat négatif au virus d’Ébola lorsqu’ils ont été testés par RCP pour une confirmation en laboratoire. Les échantillons de sang de trois patients ont été rejetés car ils ne correspondaient pas à la définition des cas suspects et deux sujets ont refusé la prise de sang. Le réseau de surveillance à l’échelle de la communauté ne s’est pas vu confier un rôle important dans la surveillance et la réaction au virus d’Ébola, comme en témoigne le fait que les volontaires de ce réseau et le personnel de santé n’avaient pas été formés aux procédures correspondantes. La crise du virus d’Ébola a suscité un intérêt public considérable en août et septembre 2014, mais cet intérêt est retombé, pour des raisons qu’il y aura lieu de déterminer officiellement. Les craintes et inquiétudes

infondées concernant le virus d'Ébola continuent à poser des difficultés, en raison du manque de connaissances approfondies sur la maladie au Ghana.

Conclusion : Le Ghana a été l'un des pionniers du déploiement de la surveillance et de la réaction intégrées aux maladies en Afrique. Pourtant, nous avons identifié des lacunes dans l'exécution pratique de ce système en relation avec le virus d'Ébola dans la région de Brong Ahafo. Afin de combler ces lacunes et de déployer efficacement le système de SRRI dans la région, il faudrait que le réseau de surveillance à l'échelle de la communauté devienne un partenaire actif de la surveillance par les centres de santé.

Translated from English version into French by Suzanne Assenat, through

TRANSLATORS WITHOUT BORDERS

Оценка полезности интегрированного надзора и реагирования на заболевания относительно случаев подозрения на вирус Эбола в регионе Бронг-Агафо, Гана

Исса Кофи, Нарти Кеннеди, Амоа Ричард, Бакан Джордж Эммануэль, Алиба Якоб, Йетли Энуама и Летса Тимоти (Issah Kofi, Nartey Kennedy, Amoah Richard, Bachan George Emmanuel, Aleeba Jacob, Yeetey Enuamah and Letsa Timothy)

КРАТКОЕ ИЗЛОЖЕНИЕ

Основание: Это исследование оценивало качество, основные и сопровождающие функции системы интегрированного надзора и реагирования на заболевания относительно 18 случаев подозрения на заболевание вирусом Эбола в регионе Бронг-Агафо, Гана.

Методы: Данные были собраны по определенным индикаторам системы надзора в случае 18 подозрений на вирус Эбола в ходе санитарно-противоэпидемиологической недели с 19 по 45, в 2014 году. Нами были проведены углубленные интервью с участием руководящих работников медицины, семи директоров и двух районных директоров сферы здравоохранения. Также мы изучили документацию, касающуюся применения основных и сопроводительных услуг и качества системы интегрированного надзора и реагирования. Также мы изучили новости сми и слухи о вирусе Эбола в обществе, а также данные записей службы надзора.

Результаты: Исследование выявило пробелы в применении системы интегрированного надзора и реагирования относительно 18 случаев подозрения на вирус Эбола. Медицинский персонал в значительной степени опирается на кровотечение как на единственный симптом, указывающий на подозрение на вирус Эбола. Двенадцать анализов крови и мазок выделений из ротовой полости тринадцатого пациента (который умер) показали отрицательный результат на вирус Эбола с использованием PCR – анализа в лабораторном подтверждении. Анализы крови трех пациентов не были учтены, так как они не подходили под описание случаев, подозрительных на вирус Эбола, а двое отказались сдавать кровь на анализ. Система общественного надзора не играет значительной роли в системе надзора и реагирования, как указывается волонтерами системы, медицинский персонал не получает обучения в этой сфере. В августе и сентябре 2014 в обществе наблюдался значительный интерес к вирусу Эбола. С тех пор интерес упал по причинам, которые требуют формального выявления. Необоснованный страх и беспокойство относительно вируса

Эбола по-прежнему представляют собой проблему в связи с недостатком знаний об этом заболевании в Гане.

ВЫВОД: Гана стала одной из первых стран в Африке, где была применена система надзора и реагирования. Несмотря на это, в регионе Бронг-Агафо были выявлены недостатки в применении системы в отношении вируса Эбола. Чтобы ликвидировать эти недостатки, система общественного надзора должна активнее сотрудничать с медицинскими учреждениями для обеспечения эффективной работы системы надзора и реагирования в этом регионе.

Translated from English version into Russian by Anna Philippova, through

TRANSLATORS WITHOUT BORDERS

Evaluación de la utilidad de la Vigilancia Integrada de las Enfermedades y Respuesta en casos sospechosos de ébola en la región de Brong Ahafo en Ghana

Issah Kofi, Nartey Kennedy, Amoah Richard, Bachan George Emmanuel, Aleeba Jacob, Yeetey Enuamah y Letsa Timothy

RESUMEN

Antecedentes: El presente estudio evaluó las funciones básicas y de apoyo y atributos de calidad del sistema de vigilancia integrada de las enfermedades y respuesta (IDSR) en 18 casos sospechosos de enfermedad por el virus del ébola (EVE) en la región de Brong Ahafo en Ghana.

Métodos: Se recolectaron datos de indicadores selectos del sistema de vigilancia con relación a 18 casos sospechosos de EVE, de las semanas epidemiológicas 19 a la 45 en el año 2014. Se condujeron entrevistas detalladas con siete directores médicos y dos directores de distrito de servicios de salud, y también se revisó la documentación sobre la implentación de funciones básicas, de apoyo y atributos de calidad del sistema IDSR. También se monitorearon las noticias en los medios y rumores sobre la EVE dentro de la comunidad y en los registros de vigilancia de las instituciones de salud.

Resultados: El estudio identificó déficits en la implementación del sistema IDSR en cuanto a los 18 casos sospechosos de EVE. El personal de la salud se basaba fuertemente en la hemorragia como único síntoma para la detección de casos sospechosos de EVE. Doce muestras de sangre y un frotis de secreción bucal del paciente número trece (que falleció) fueron negativas para EVE cuando se utilizó la técnica PCR para la confirmación de laboratorio. Se descartaron muestras de sangre de tres pacientes, ya que no cumplían con la definición para casos sospechosos, y dos pacientes se rehusaron a que se les tomen muestras de sangre. No se le ha dado importancia al sistema de vigilancia epidemiológica basada en la comunidad (VEBC) en la vigilancia y respuesta de la EVE, tal como lo demuestra el hecho de que voluntarios y trabajadores de la VEBC no recibieron entrenamiento sobre dichos procesos. Hubo un intenso interés público en la EVE en agosto y septiembre de 2014. Dicho interés ha menguado por razones que todavía tienen que verificarse. El miedo sin fundamento y la ansiedad sobre la EVE todavía siguen siendo un desafío debido a la falta de conocimiento detallado sobre la enfermedad en Ghana.

CONCLUSION: Ghana ha sido uno de los pioneros en la implementación del sistema IDSR en África. A pesar de ello, se han identificado déficits en la implementación del sistema IDSR con respecto a la EVE en la región de Brong

Ahafo. Para abordar dichos déficits, el sistema de VEBC tiene que asociarse de manera activa con la vigilancia en los centros de salud para lograr una IDSR efectiva en la región.

Translated from English version into Spanish by Maria Alejandra Aguada, through

TRANSLATORS
WITHOUT BORDERS
